# Mycotoxin Contamination in Sugarcane Grass and Juice: First Report on Detection of Multiple Mycotoxins and Exposure Assessment for Aflatoxins B_1_ and G_1_ in Humans

**DOI:** 10.3390/toxins8110343

**Published:** 2016-11-18

**Authors:** Mohamed F. Abdallah, Rudolf Krska, Michael Sulyok

**Affiliations:** 1 Department of Forensic Medicine and Toxicology, Faculty of Veterinary Medicine, Assiut University, Assiut 71515, Egypt; mohamed.fathi@vet.au.edu.eg; 2 Center for Analytical Chemistry, Department of Agrobiotechnology (IFA-Tulln), University of Natural Resources and Life Sciences, Vienna (BOKU), Konrad Lorenz Str 20, Tulln A-3430, Austria; rudolf.krska@boku.ac.at

**Keywords:** mycotoxins, sugarcane, sugarcane juice, aflatoxins, LC-MS/MS, exposure assessment

## Abstract

This study was conducted to investigate the natural co-occurrence of multiple toxic fungal and bacterial metabolites in sugarcane grass and juice intended for human consumption in Upper Egypt. Quantification of the target analytes has been done using the “dilute and shoot” approach followed by liquid chromatography-tandem mass spectrometry (LC-MS/MS). A total number of 29 and 33 different metabolites were detected in 21 sugarcane grass and 40 juice samples, respectively, with a trend of concentrations being higher in grass than in juice. Among the regulated mycotoxins, only aflatoxin B_1_ (AFB_1_) and aflatoxin G_1_ (AFG_1_) were detected. The prevalence of AFB_1_ was in 48% of grass samples and in 58% of juice with a maximum concentration of 30.6 μg/kg and 2.10 μg/kg, respectively. AFG_1_ was detected in 10% of grass samples (7.76 μg/kg) and 18% of juice samples (34 μg/kg). Dietary exposure was assessed using a juice frequency questionnaire of adult inhabitants in Assiut City. The assessment revealed different levels of exposure to AFB_1_ between males and females in winter and summer seasons. The estimated seasonal exposure ranged from 0.20 to 0.40 ng/kg b.w./day in winter and from 0.38 to 0.90 ng/kg b.w./day in summer.

## 1. Introduction

Sugarcane, *Saccharum officinarum*, is a tropical tall perennial grass cultivated in several countries of the world. In Africa, sugarcane is the second most cultivated crop after cassava, where Egypt maintains the second position after South Africa [1]. In Egypt, around 97% of the total sugarcane production, 16 million tons in 2014, is cultivated in the upper part of the country [2,3]. From the economical point of view, sugarcane is an important cash crop beside cotton and a major contributor of income and employment for farmers. The plant has a high sucrose and low fiber content and is used mainly for raw sugar and molasses production (brownish-black viscous syrup known as black honey in Egypt), in addition to the grass left over or bagasse, which is used as an animal feed supplement or fertilizer. Furthermore, several secondary industries such as vinegar, alcohol, chipboard, paper, some chemicals, plastics, paints, fiber, insecticides and detergents are based on sugarcane and its wastes [2,4,5,6]. It has been estimated that 80% of the world’s sugar comes from sugarcane [7,8], where Brazil and India are the largest producers worldwide [4,6]. The annual consumption of sugar in Egypt in 2010 was estimated to be 34 kg per capita [7]. 

During the harvesting time, chewing raw sugarcane is a common practice. In addition, sugarcane juice is considered the most popular fresh juice in Egypt, with cane juice shops spreading through all the Egyptian cities. Indian and Pakistani people share the same habit with Egyptians regarding chewing raw sugarcane and consumption of juice [9,10]. Apart from its sweet taste and being a source of energy and minerals, sugarcane juice consumption, in traditional medicine, helps in the treatment of many diseases such as jaundice, kidney stones, urogenital tract infections, and in lowering blood pressure, and healing dermal wounds; it is also reported as a natural antioxidant under various experimental conditions [10,11]. 

According to the Food and Agriculture Organization, 25% of the world’s crops are contaminated by fungal toxic metabolites [12]. Sugarcane is a suitable host for many saprophytic fungi, especially the aflatoxigenic ones that belong to the *Aspergillus* species [13]. Products of secondary fungal metabolism, some of them being toxic and thus termed mycotoxins, can be formed either in the field and/or during storage. The most significant mycotoxins in terms of food and feed safety are aflatoxins, ochratoxin A, fumonisins, zearalenone, trichothecenes, and ergot alkaloids that have carcinogenic, mutagenic, teratogenic, cytotoxic, neurotoxic, nephrotoxic, estrogenic, dermotoxic, and immunotoxic effects [12,14,15,16]. 

Ingestion of contaminated food is the principal route for human exposure to mycotoxins [17]. One of the most important aspects in the risk analysis of food contaminants is to determine the degree of human exposure [18]. In the case of mycotoxins, this exposure is generally assessed by taking into account data on mycotoxin occurrence in foodstuffs as well as data on the dietary consumption of the concerned population [19,20], although analytical methods for determination of individual exposure by analysis of biofluids have recently been developed [21,22,23,24].

There are some methods used to assess dietary consumption, generally known as market basket, 24 h dietary recall and food record methods, food frequency methods or dietary history [25]. The degree of exposure is measured in terms of probable daily intake (PDI) per unit of body weight, and is generally expressed in ng/kg of body weight (b.w.) per day. Afterwards, the PDI value is compared with the tolerable daily intake (TDI) which is determined by certain toxicological studies for risk analysis. Several exposure and risk assessment studies for mycotoxins in different food commodities were carried out during the last few years [25,26,27,28,29,30,31,32]; nevertheless, no exposure assessment for mycotoxins from contaminated sugarcane grass and juice has been conducted yet.

The growth of toxicogenic fungi in a sugarcane crop field was documented in numerous studies [13,33]. However, these previous studies discussed only the isolation of different fungal species from the plant and the prevalence of their mycotoxins after inoculation of the isolated fungi in culture media without determining the natural (co-)occurrence of the produced mycotoxins. Ahmed et al. (2010) screened the contamination of sugarcane juice sold in Pakistan with several mycoflora including *Aspergillus flavus*, *A. fumigatus*, and *A. niger* [9], while Hariprasad et al. (2015) investigated the natural aflatoxin uptake by sugarcane from contaminated soil and its persistence in sugarcane juice and jiggery (the natural sweetener made by concentrating the sugarcane juice) using thin layer chromatography and ELISA [34]. To the best of the authors’ knowledge, no reports have been published for the natural occurrence of multiple mycotoxins in cane grass and juice. Moreover, no regulations in Egypt or other countries for mycotoxins in this commodity have been established so far. Therefore, it was worthwhile to perform this survey to screen for a wide range of (toxic) fungal metabolites in sugarcane grass and juice sold in Assiut City, Egypt, using liquid chromatography-tandem mass spectrometry (LC-MS/MS) with an estimation of the seasonal human exposure to mycotoxins from sugarcane juice in order to evaluate the possible health risks.

## 2. Results

### 2.1. Occurrence of Fungal and Bacterial Metabolites in Sugarcane Grass and Juice Samples

Overall, 29 different metabolites in cane grass and 33 in cane juice were quantified and, on average, 14 and 13 different metabolites were detected per sample, respectively. In both matrixes, 20 similar or shared metabolites were detected, including, aflatoxin B_1_ and G_1_, averufin, 3-nitropropionic acid, kojic acid, asperglaucide, asperphenamate, and emodin, as presented in Table 1. None of the other mycotoxins addressed by regulatory limits in the European Union (EU) have been positively identified in any of the investigated samples.

Nine different metabolites were detected exclusively in grass and another 13 metabolites were only found in juice. However, the concentrations of all the shared metabolites were higher in grass than in juice; the prevalence was variable between both commodities. As an example, the maximum concentrations of aflatoxin B_1_ and G_1_ (30.6 and 7.76 μg/kg) in grass were higher than in juice (2.10 and 1.34 μg/kg), while the prevalence of these mycotoxins in grass (48% and 10%) was lower than in juice (58% and 18%), respectively. Data on the maximum concentration of all evaluated mycotoxins in each commodity, as well as the related median, mean and apparent recovery in the positive samples, are compiled in Table 2 and Table 3.

Asperphenamate was detected in all grass and juice samples (100%). The other most prevalent metabolites in grass were emodin (100%), tryptophol (95%), citreorosein (86%), iso-rhodoptilometrin (81%), *N*-Benzoyl-Phenylalanine (81%), kojic acid (76%), and ilicicolin B (67%), while tryptophol (100%), emodin (95%), citreorosein (88%), ilicicolin B (88%), averufin (68%), and iso-rhodoptilometrin (68%) were the most frequently occurring ones in cane juice. 

Kojic acid was detected in 3% of juice, which appeared to be much lower in comparison with those found in grass samples, in 76% of the analyzed samples. In addition to aflatoxins as important toxic metabolites, 3-nitropropionic acid (3-NPA) was detected in both grass and juice; however, the frequencies and concentrations were lower in juice (Table 2 and Table 3). The co-occurrence of *Aspergillus flavus* metabolites (AFB_1_, averufin, 3-NPA, and kojic acid) was detected in ≥28% of grass, while no juice samples were co-contaminated with all of these metabolites.

It was noticeable that the prevalence of the non-shared metabolites in grass was higher than in juice, in which four metabolites occurred in more than half of the samples, cyclo (L-Pro-L-Val) (100%), cyclo (L-Pro-L-Tyr) (67%), *N*-Benzoyl-Phenylalanine (81%), and physcion (81%)), while in juice only one metabolite occurred in more than the half the samples, versicolorin C in 73% of juice.

### 2.2. Exposure Assessment

The average daily consumption of juice for adult females (*n* = 90) and males (*n* = 91) was estimated in winter at 34.1 and 64.2 mL/day, and in summer at 66.3 and 150.4 mL/day, respectively. Due to the significant variability in juice consumption patterns between males and females in winter and summer seasons, each group for each season was considered alone for the exposure assessment.

The probable daily intakes (PDI) were obtained by integration of the results of analyzed mycotoxins with the juice consumption estimation with a body weight of 70 kg for adults. The PDI values (ng/kg) for AFB_1_ and AFG_1_ (Table 4) were calculated according to the following equation: PDI = (C × K)/b.w., where C is the mean content of a mycotoxin (μg/kg) and K is the average consumption of the commodity (mL/day). Mean values of all juice samples were 0.42 μg/kg for AFB_1_ and 0.05 μg/kg for AFG_1_.

Due to the fact that aflatoxins (AFs) are potent liver carcinogens [35], there is no TDI for AFs (no safe level can be established as AFs can induce cancer even at very low doses). Therefore, most agencies, including the Joint Expert Committee on Food Additives and the US Food and Drug Administration, have not set a TDI for AFs. Of note, the obtained rate of exposure is expected be higher for children (due to the difference in body weight) and for adult persons (in case of a high rate of consumption). 

As shown above in Table 4, the PDI values appear to be more than double during summer and male inhabitants are likely to be more exposed throughout the year than females.

## 3. Discussion

The sugarcane crop and its by-products have a great agro-industrial and economic value. Upper Egypt governorates are the main home to sugarcane cultivation and its industry in Egypt, particularly in the governorates of Sohag, Qena and Aswan [36]. The choice of Assiut City, not a major state for sugarcane cultivation in Egypt, was based on the availability of the samples and collection of data by the authors. 

During harvest time, cutting of the sugarcane stalk disrupts the physiology of the plant and acts as a portal of entry for pathogenic bacteria and fungi. Some of these microorganisms, under elevated temperatures and high humidity, use the sugar as a source of energy for growth and produce different metabolic by-products that, on one hand, may cause processing problems in the mill and refinery and, on the other hand, can be toxic to animals and humans [33,37]. Red rot, smut, wilt and sett rot are the most important fungal diseases affecting sugarcane agriculture and causing remarkable economic losses [38,39,40]. This necessitates a detailed investigation of the probable effect of mycotoxins on the sugar industry and the other by-products such as alcohol as one of the main products derived from sugarcane. 

In the light of the aforementioned results, the level of AFB_1_ in sugarcane was higher than the highest maximum level regulated by the EU (12 μg/kg for unprocessed almonds, pistachios, and apricot kernels) [41] and Egyptian standards for peanuts intended for human consumption (5 μg/kg) [42]. AFs in sugarcane crops can be derived from two sources, either through the natural AF uptake from contaminated soil in the field [34] or from fungal attack to the outer fiber layer, especially after insect invasion or other parasites pre- or post-harvest [43,44,45]. Also, it is worth mentioning that chewing raw sugarcane is a common practice, especially during the harvesting time. However, no regulations for AFs in fruit or crop juices have been set. Recently, Hariprasad et al. (2015) detected higher concentrations of AFB_1_ in sugarcane juice, 0.5–6.5 μg/kg, collected from local vendors in India [34]. In a previous study, AFB_1_ was detected in mango (0.03–0.72 μg/L), guava (0.04–0.20 μg/L), apple (0.03–0.07 μg/L) and orange (0.006–0.07 μg/L) juices collected from local markets of six governorates in Egypt. The maximum concentration of AFG_1_ in mango, orange, guava and apple nectar was 0.08, 0.21, 0.34, and 0.55 μg/L, respectively [46]. The detection of multiple mycotoxins in different Egyptian canned fruits juices and beverages was also investigated by Abdel-Sater et al. (2001) using thin layer chromatography. Total AFs (AFB_1_ and AFG_1_) in apple beverages ranged from 20–30 μg/L and AFB_1_ in guava juice was at a concentration of 12 μg/L [47].

Two hypotheses for sugarcane juice contamination with the detected metabolites including AFs were developed; (1) contaminated sugarcane stems used for juice production; (2) contamination of the instruments used in juicing. A reported case in China showed that 3-NPA was the main causative agent of acute poisoning after consumption of moldy sugarcanes [48]. Moreover, 88 persons died out of the 884 exposed to moldy sugarcane during the period of 1972 until 1988 [49]. In animals the systemic administration of 3-NPA is suspected to cause Huntington's disease–like symptoms [50]. In a survey from Argentina in 2011 and 2014, the maximum concentration in native grass for grazing cattle ranged from 28.8–120 μg/kg [51] which is lower than the present report; however, higher concentrations were detected by Ezekiel et al. (2012) in poultry feed from Nigeria (up to 947 μg/kg) [52]. Therefore, the toxic effect of 3-NPA may affect humans through chewing raw sugarcane grass while it may affect animals through feeding on grass left over or on the bagasse of sugarcane. 

Sugarcane juice consumption is a daily habit in Egypt, continuing throughout the year. However, the consumption rate greatly increases during the summer months. A common approach to estimate mycotoxin exposure is generally obtained through the combination of contamination results with the consumption data. So far, to the best of the authors’ knowledge, there are no available data on dietary exposure to mycotoxins from sugarcane juice and/or juice consumption in Egypt or anywhere else of the world.

There are some methods used to assess dietary intake such as the food frequency questionnaire. This approach was adopted to assess the dietary exposure to different mycotoxins by several previous studies in Brazil, Spain, Japan, Malaysia [25,28,30,32,53]. In this study, the juice frequency questionnaire (online and paper based) was chosen. The participants, 91 males and 90 females, were of different ages (18–65 years old). The difference in PDI values between males and females is related to the juice intake rates. The reason may be connected with the nature of Egyptian society where males are more likely to be in contact with streets and juice shops. Also, the difference between winter and summer is due to climatic factors such as temperature. 

Extensive AF exposure studies with estimation of PDI values have been reported, although little is available in the literature concerning the AFB_1_ contamination in juices or similar beverages [46,47,54]. In Egypt, the PDI values of AFB_1_ from contaminated corn-based snacks for adults ranged from 0.42 to 11.30 ng/kg b.w./day; however, the consumption data (50 g/day) were estimated by the authors based on the content packaging of the commercial product and PDI values were estimated with the mean value of positive samples [55]. In another study from Cairo using the same foodstuffs, the probable daily intake of aflatoxin B_1_ was 3.69 ng/kg b.w./day, although the value was calculated with the mean of the positive samples as well [56]. The same author in another survey, El-Sawi in 2006, estimated the PDI of AFB_1_ (0.097 ng/kg b.w./day) from corn using the mean values of contaminated samples in the calculations [57]. The mean PDI values of AFB_1_ were assessed worldwide: Korea (1.19 and 5.79 ng/kg b.w./day from rice) [27], Malaysia (24.3 to 34.0 ng/kg b.w./day and for total AFs was 28.81 to 58.02 ng/kg b.w./day from 236 individual food composites consisting of 38 different types of foods) [53], Lebanon (0.63–0.66 ng/kg b.w./day from Lebanese diets) [58], Japan (0.003 to 0.004 ng/kg b.w./day, from different 24 foods categories including peanuts) [32], and Brazil (2.3 to 4.1 ng/kg b.w./day of AFs from peanuts for high consumers) [28].

Consequently, the estimated PDI levels of AFB_1_ and AG1 for the Egyptian population from sugarcane juice consumption in both seasons may contribute to a public health implication due to the fact that even low levels of AFs contamination, which might fall with the permissible limits, can lead to haptic cancer in the long run, in addition to the inevitable exposure to AFs from other contaminated food commodities. Also, serious health implications can be expected in case of high rates of juice consumption.

## 4. Conclusions

The study aimed to detect multiple mycotoxins occurring naturally in the sugarcane crop and juice for the first time. Indeed, the present work will open the door for further studies on the occurrence of more mycotoxins in this important economic crop. Furthermore, the presence of these metabolites either in their present (parent) form or in a modified form in the secondary products, which are based on sugarcane products such as raw sugar, vinegar, alcohol, chipboard, paper, some chemicals, plastics, paints, fiber, insecticides and detergents and molasses, cannot be excluded.

The study sheds light on the non-negligible doses of aflatoxins through the consumption of juice in Upper Egypt, especially in the summer. However, the survey was limited to 181 persons in Assiut City. Also, it should be noted that chewing raw sugarcane grass is a common practice during the harvesting time which indicates an additional source of mycotoxin exposure. These first data for exposure warrant further larger-scale multi-mycotoxin-based studies aimed at providing a comprehensive assessment in other Egyptian cities as the consumption may vary among different social groups within Egypt. Due to the seasonal fluctuation of the fungal species [33,59,60] and the difference in consumption rates of juice as shown in the present survey, follow-up detection studies in summer and in other cities of the country are urgently needed to provide better insights regarding the number of contaminating mycotoxins and their quantities in both grass and juice. 

It is highly recommended that a study for risk assessment in different foodstuffs be performed to estimate the contribution of sugarcane juice in comparison with other foods and beverages in the Egyptian diet. This report will assist the other countries where sugarcane grass cultivation and juice consumption are common to assess the occurrence of different mycotoxins in these commodities and the consequent possible health risks. Another important point needs to be mentioned herein regarding the contribution of grass left over from sugarcane in animal feed, as it is a very cheap supplement and a rich source of fiber. A detailed investigation must be conducted to detect the contaminating mycotoxins and the possibility of co-exposure in producing farm animals.

In order to limit the fungal attack and the potential production of toxic metabolites, appropriate pre-harvest precautions should be applied. Considering the juice, it should be produced in a hygienic media after a proper cleaning of the grass, especially the outer external cortex of the plant, as well as the wringers or the machine used for juicing, which can be a source of contamination.

## 5. Materials and Methods

### 5.1. Sample Collection 

Sugarcane stem (*n* = 21) and fresh sugarcane juice (*n* = 40, each 400 mL) samples were randomly purchased from several shops as would be done by a consumer from local vendors and juice shops, respectively in January 2016 from Assiut City, Assiut Governorate, Egypt. After collection, juice samples were kept at −20 °C until LC-MS/MS analyses. 

### 5.2. Sample Treatment, Extraction and Mycotoxins Analysis

#### 5.2.1. Chemicals and Reagents

Acetonitrile (LC gradient grade) were purchased from VWR International (Leuven, Belgium), methanol (LC gradient grade) and glacial acetic acid (p.a.) from Merck (Darmstadt, Germany), and ammonium acetate MS grade) from Sigma-Aldrich (Vienna, Austria). Water was purified successively by reverse osmosis and an Elga Purelab ultra analytic system from Veolia Water (Bucks, UK) to 18.2 MΩ. Standards of fungal and bacterial metabolites were obtained either as gifts from various research groups or from the following commercial sources: Romer Labs^®^ Inc. (Tulln, Austria), Sigma-Aldrich (Vienna, Austria), Iris Biotech GmbH (Marktredwitz, Germany), Axxora Europe (Lausanne, Switzerland) and LGC Promochem GmbH (Wesel, Germany), Enzo Life Sciences (Lausen, Switzerland), BioAustralis (Smithfield, Australia), AnalytiCon Discovery (Potsdam, Germany) and Toronto Research Chemicals (Toronto, ON, Canada).

#### 5.2.2. Extraction and Estimation of Apparent Recoveries

For juice, 2 mL of each sample were extracted with 2 mL of the extraction solvent (acetonitrile/water/acetic acid, 79/20/1, *v*/*v*/*v*), centrifuged for 2 min at 4000 RPM at room temperature, and diluted 1:1 with dilution solvent (acetonitrile/water/acetic acid, 20/79/1, *v*/*v*/*v*). 

For sugarcane grass, each collected stem (with no water content either the external fiber layer or collected fresh leftover or bagasse from shops and all were dried in oven at Romer lab, Tulln for 24 h at 30 °C) was ground into very small pieces (<1 mm) and 40 mL of the extraction solvent were added to 2.5 g of each sample. Afterwards, samples were shaken for 90 min at 180 RPM at room temperature, and then diluted 1:1 with dilution solvent. The volume of injection for juice and sugarcane grass was 5 μL of the diluted raw extract without further manipulation according to Sulyok et al. (2006) [61].

### 5.3. LC-MS/MS Parameters

The samples were analyzed using a dilute and shoot approach as described before in the literature [62]. Briefly, an Agilent 1290 Series HPLC System (Agilent, Waldbronn, Germany) coupled to a QTrap 5500 used in connection with a Turbo Ion Spray ESI source (Sciex, Foster City, CA, USA) has been used in connection with a Gemini^®^ C18-column, 150 × 4.6 mm i.d., 5 μm particle size, protected by a C18 security guard cartridge, 4 × 3 mm i.d. (all from Phenomenex, Torrance, CA, USA). A methanol/water gradient containing 1% acetic acid and 5 mM NH_4_Ac was used at 1 mL/min. Data acquisition was performed in the scheduled multiple reaction monitoring (sMRM) mode in both positive and negative polarity using two separate chromatographic runs per sample. External calibration was performed using serial dilutions of a multi-component working standard. Results were corrected for apparent recoveries that have been determined by spiking five individual samples of each matrix. 

### 5.4. Sugarcane Juice Consumption Data

Due to lack of data for juice consumption in Egypt, juice frequency questionnaires (online and paper based) were filled in by different inhabitants in Assiut City. The participants were requested to answer the monthly consumption rate in winter and summer seasons. In order to know the exact amount per mL, two different sizes of juice cups, large and small, as sold by local shops in Egypt, were included in the questionnaires. The large cup is equal to 350 mL while the small one is 250 mL. Additional information such as age, level of education, and distance from the nearest juice shop were also documented. The total number was 181 adults inhabitants (18–65 years) of Assiut city participated in the questionnaires, females (*n* = 90) and males (*n* = 91). 

## Figures and Tables

**Table 1:** Overview of the detected analytes in sugarcane grass and juice samples.

Metabolites in Both	Metabolites only in Cane Grass	Metabolites only in Cane Juice
3-Nitropropionic acid	Alternariolmethylether	Aspinolid B
Aflatoxin B_1_	Brevianamid F	Chlorocitreorosein
Aflatoxin G_1_	Cyclo (L-Pro-L-Tyr)	Fusapyron
Agroclavine	Cyclo (L-Pro-L-Val)	Fusaric acid
Ascochlorin	Cytochalasin D	Gibberellic acid
Asperglaucide	Ilicicolin E	Griseofulvin
Asperphenamate	Macrosporin	Integracin A
Averufin	*N*-Benzoyl-Phenylalanine	Integracin B
Berkedrimane B	Physcion	Monocerin
Citreorosein	-	Nidurufin
Emodin	-	Versicolorin A
Ilicicolin B	-	Versicolorin C
Iso-Rhodoptilometrin	-	Xanthotoxin
Kojic acid	-	-
Norlichexanthone	-	-
Oxaline	-	-
Penicillic acid	-	-
Quinolactacin A	-	-
Skyrin	-	-
Tryptophol	-	-

**Table 2:** Overview on the occurrence, concentrations and performance characteristics of the analytical method for the detected analytes in natural sugarcane grass samples.

Concentration of Positive Samples (μg/kg)									
Detected Analytes	P/N	Prevalence	Median	Mean	Minimum	Maximum	R_a_	LOD ^a^	LOQ ^b^
3-Nitropropionic acid	13/21	62%	5.48	27.5	<LOQ	193	81.4%	0.9	2.8
Aflatoxin B_1_	10/21	48%	11.7	13.6	<LOQ	30.6	53.4%	2.8	9.2
Aflatoxin G_1_	2/21	10%	5.10	5.10	<LOQ	7.76	41.6%	2.2	7.4
Agroclavine	2/21	10%	161	161	<LOQ	300	71.4%	13	44
Alternariolmethylether	9/21	43%	0.23	0.45	<LOQ	1.26	60%	0.05	0.2
Ascochlorin	6/21	29%	2.63	11.5	0.8	55.4	80.2%	0.1	0.4
Asperglaucide	9/21	43%	0.41	0.66	<LOQ	2.07	54.6%	0.1	0.4
Asperphenamate	21/21	100%	17.2	283	1.81	3998	87%	0.08	0.3
Averufin	11/21	52%	0.36	0.48	<LOQ	1.91	85.6%	0.05	0.2
Berkedrimane B	4/21	19%	4.39	18.3	<LOQ	64.2	54.2%	0.2	0.7
Brevianamid F	4/21	19%	13.8	13.5	6.30	20.0	45%	0.8	2.7
Citreorosein	18/21	86%	8.33	22.1	<LOQ	229	163%	0.8	2.7
Cyclo (L-Pro-L-Tyr)	14/21	67%	7.99	15.4	<LOQ	55.3	90.4%	1.7	5.6
Cyclo (L-Pro-L-Val)	21/21	100%	16.1	31.6	<LOQ	198	94.6%	1	3.4
Cytochalasin D	2/21	10%	71.5	71.5	5.40	137	80.4%	0.5	1.8
Emodin	21/21	100%	4.31	7.37	<LOQ	34.1	121%	0.2	0.8
Ilicicolin B	14/21	67%	28.8	43.6	5.44	141	42.2%	1.3	4.2
Ilicicolin E	1/21	5%	0.39	0.39	0.39	0.39	76.8%	0.1	0.3
Kojic acid	16/21	76%	4160	20,176	617	117,815	99.6%	75.8	250
Iso-Rhodoptilometrin	17/21	81%	0.89	6.00	0.19	72.9	110%	0.04	0.1
Macrosporin	5/21	24%	0.08	5.91	<LOQ	29.2	94.2%	0.02	0.05
*N*-Benzoyl-Phenylalanine	17/21	81%	8.29	66.1	<LOQ	856	93.6%	2.3	7.4
Norlichexanthone	8/21	38%	26.6	149.7	<LOQ	1025	65%	1.1	3.6
Oxaline	2/21	10%	13.0	13.0	<LOQ	25.7	70%	0.17	0.57
Penicillic acid	10/21	48%	70.5	333	13.2	2683	97%	2.5	8.1
Physcion	17/21	81%	60.4	58.2	<LOQ	73.5	98.4%	39	130
Quinolactacin A	7/21	33%	0.51	0.74	0.12	2.33	64%	0.03	0.1
Skyrin	4/21	19%	2.50	2.67	<LOQ	4.71	79.4%	0.8	2.7
Tryptophol	20/21	95%	318	887	27.6	4370	64.4%	7.08	23

Calculation of mean, median and range values was based on positive samples. P/N, number positive samples over the number of total samples; R_a_, apparent recovery; ^a^ LOD, limit of detection; ^b^ LOQ, limit of quantification.

**Table 3:** Overview on the occurrence, concentrations and performance characteristics of the analytical method for the detected analytes in sugarcane juice samples.

Concentration of Positive Samples (μg/kg)									
Detected Analytes	P/N	Prevalence	Median	Mean	Minimum	Maximum	R_a_	LOD ^a^	LOQ ^b^
3-Nitropropionic acid	12/40	30%	2.84	3.58	<LOQ	13.6	67.6%	0.2	0.7
Aflatoxin B_1_	23/40	58%	0.56	0.72	<LOQ	2.10	73.9%	0.14	0.4
Aflatoxin G_1_	7/40	18%	0.10	0.30	<LOQ	1.34	62.4%	0.06	0.2
Agroclavine	8/40	20%	3.05	3.96	<LOQ	9.49	68%	0.004	0.01
Ascochlorin	18/40	45%	0.22	0.28	<LOQ	0.63	78.4%	0.1	0.3
Asperglaucide	7/40	18%	0.03	0.21	<LOQ	0.83	93.6%	0.01	0.02
Asperphenamate	40/40	100%	5.23	12.5	0.65	91.4	96.7%	0.2	0.6
Aspinolid B	1/40	3%	7.92	7.92	7.92	7.92	96.5%	0.04	0.13
Averufin	27/40	68%	0.12	0.13	<LOQ	0.32	85.2%	0.06	0.2
Berkedrimane B	26/40	65%	0.41	5.75	<LOQ	41.9	100%	0.004	0.01
Chlorocitreorosein	19/40	48%	3.26	8.65	<LOQ	51.9	102%	0.8	1.2
Citreorosein	35/40	88%	1.28	1.99	<LOQ	12.6	107%	0.1	0.4
Emodin	38/40	95%	0.45	0.82	<LOQ	3.90	92.9%	0.01	0.02
Fusapyron	7/40	18%	0.98	7.49	<LOQ	44.4	71.3%	0.1	0.4
Fusaric acid	12/40	30%	67.9	258	25.4	2214	92.4%	1.33	4.39
Gibberellic acid	10/40	25%	2.30	18.7	<LOQ	134	128%	0.2	0.7
Griseofulvin	2/40	5%	0.36	0.36	0.20	0.51	97.5%	0.05	0.17
Ilicicolin B	35/40	88%	0.59	2.44	<LOQ	17.6	52.6%	0.07	0.2
Kojic acid	1/40	3%	208	208	208	208	62.7%	13.8	45.6
Integracin A	2/40	5%	1.73	1.73	1.60	1.87	67.1%	0.3	0.9
Integracin B	2/40	5%	1.12	1.19	<LOQ	1.97	64.2%	0.2	0.6
Iso-Rhodoptilometrin	27/40	68%	0.03	0.06	<LOQ	0.36	103%	0.01	0.02
Monocerin	5/40	13%	0.28	1.26	<LOQ	4.97	100%	0.02	0.08
Nidurufin ^c^	14/40	35%	0.10	0.15	0.001	0.61	n.d ^e^	-	-
Norlichexanthone	11/40	28%	0.84	1.67	<LOQ	6.05	82.5%	0.03	0.10
Oxaline	1/40	3%	1.61	1.61	1.61	1.61	64.6%	0.10	0.32
Penicillic acid	16/40	40%	19.9	45.7	3.02	212	77.2%	0.2	0.7
Quinolactacin A	22/40	55%	0.03	0.14	0.005	1.40	80.2%	0.001	0.004
Skyrin	7/40	18%	0.17	0.35	0.05	1.60	79.8%	0.03	0.1
Tryptophol	40/40	100%	58.4	110	<LOQ	581	54.9%	0.5	1.8
Versicolorin A	17/40	43%	0.05	0.06	<LOQ	0.23	80.7%	0.02	0.06
Versicolorin C ^d^	29/40	73%	7.74	11.31	1.55	53.2	n.d ^e^	-	-
Xanthotoxin	4/40	10%	2.28	1.99	<LOQ	3.32	94.6%	0.03	0.1

Calculation of mean, median and range values was based on positive samples. P/N, number positive samples over the number of total samples; R_a_, apparent recovery; ^a^ LOD, limit of detection; ^b^ LOQ, limit of quantification; ^c^ No standard available, estimation of concentration based on response and recovery of averufin; ^d^ No standard available, estimation of concentration based on response and recovery of versicolorin A; ^e^ n.d.: not determined.

**Table 4:** The probable daily intakes of AFB_1_ and AFG_1_ from sugarcane juice in Assiut City.

Season	Winter		Summer	
Mycotoxin/Gender	Females	Males	Females	Males
AFB_1_ (ng/kg)	0.20	0.38	0.40	0.90
AFG_1_ (ng/kg)	0.02	0.05	0.05	0.11
